# The Gravitation of the Moon Plays Pivotal Roles in the Occurrence of the Acute Myocardial Infarction

**DOI:** 10.4137/EHI.S900

**Published:** 2008-10-31

**Authors:** Ryotaro Wake, Junichi Yoshikawa, Kazuo Haze, Shinichiro Otani, Takayoshi Yoshimura, Iku Toda, Masaki Nishimoto, Takahiko Kawarabayashi, Atsushi Tanaka, Kenei Shimada, Hidetaka Iida, Kazuhide Takeuchi, Minoru Yoshiyama

**Affiliations:** 1 Department of Internal Medicine and Cardiology, Graduate School of Medicine, Osaka City University, Osaka, Japan; 2 Department of Cardiology, Osaka Hospital of Japan Seafarers Relief Association, Osaka, Japan; 3 Department of Cardiology, Osaka City General Hospital, Osaka, Japan; 4 Department of Internal medicine, Tane General Hospital, Osaka, Japan; 5 Department of Cardiology, Ikuwakai Memorial Hospital, Osaka, Japan; 6 Division of Cardiology, Bell Land General Hospital, Sakai, Japan; 7 Department of Internal Medicine, Izumi City Hospital, Izumi, Osaka, Japan; 8 Department of Internal Medicine, Division of Cardiology, Higashisumisyoshi Morimoto Hospital, Osaka, Japan; 9 Department of Cardiovascular Medicine, Wakayama Medical University, Wakayama, Japan; 10 Department of Cardiology, Tsukazaki Hospital, Hyougo, Japan

**Keywords:** acute myocardial infarction, biological clock, the gravitation of the moon, lunar cycle

## Abstract

Acute myocardial infarction (AMI) is a social burden. However, being able to predict AMI could lead to prevention. A previous study showed only the relation between the lunar phase and the occurrence of AMI, but the period it takes for the moon to orbit around the earth and the period of the lunar phase differ. This study investigated the effect of the gravitation of the moon on AMI. Data was comprised of 1369 consecutive patients with first AMI at 5 hospitals from October, 1984 to December, 1997. The universal gravitation of the moon was calculated and compared to the earth onset time of AMI. Universal gravitation of the moon was derived by G*m/d^2^ (G: universal gravitation constant, m: the mass of the moon, d: the distance between the center of the moon and the center of the earth). The relationship between m/d^2^ and the cases of AMI was determined. There was an increase in cases, when there is a distance of more than 399864 km from the center of the earth to the center of the moon. The gravitation of more than 399864 km was determined to be weaker gravitation. It is confirmed that the number of AMI patients significantly increases at weaker gravitation periods in this multicenter trial. In conclusion, these results suggest that the gravitation of the moon may have an influence on the occurrence of AMI.

## Introduction

**Figure 1.**
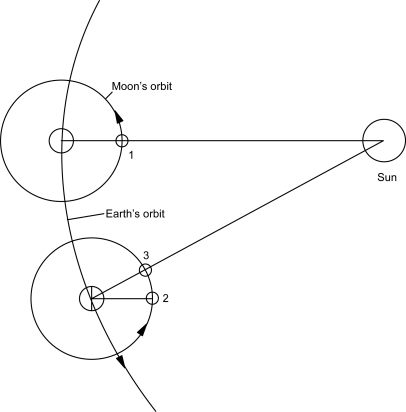
The Figure shows the relation among the earth, the moon and the sun. The synodic month (1→3) is longer than the sidereal month (1→2), because the Earth moves onwards in its orbit in the meantime.

A number of studies have reported a daily and yearly variation in the occurrence of AMI. (Hjalmarson, et al. 1989; Kloner, Poole, and Perritt, 1999; Muller, et al. 1985; Willich, et al. 1989) It has been shown that the circadian variation of AMI is the result of an increase in the incidence of plaque rupture during the morning hours. (Tanaka, et al. 2004) These studies have shown a relation between the solar motion and the occurrence of AMI. Less information is available regarding the effect of the moon on coronary artery disease, although the lunar cycle has played many important roles in our lives from ancient times, for example, the first calendar was made on the basis of the moon. Previous reports have shown lunar phases do not relate to the occurrence of AMI. (Alves, et al. 2003; Eisenburger, et al. 2003) However, the period it takes the moon to orbit around the earth and the period of the phase of the moon are different. These periods are referred to as the sidereal month and synodic month, respectively. One sidereal month is 27.32 days, and after that time, the moon has returned to the same point in the heavens. The synodic month is 29.53 days, the time after the moon returns to exactly the same position relative to the sun. The moon moves in an easterly direction relative to the earth, by 360°/29.53 = 12.2° each day, and relative to the earth, by 360°/27.32 = 13.2°. The difference between the sidereal and synodic daily motion of the moon is equal to the daily motion of the sun (Fig. 1). This becomes clear that the daily motion is nothing other than the angular velocity in astronomical units. A previous report has shown that a blind man has a circadian rhythm based on the motion of the moon. (Miles, Raynal, and Wilson, 1977) As well as the sun, the moon may control our biological clock. The study of the timing and activity at occurrence of AMI may provide clues to preventive measures. There have, however, been few studies concerning the gravitation of the moon. The aim of this study was to investigate the relation between the occurrence of AMI and the gravitation of the moon.

## Methods

### Subjects

Our study population was comprised of 1369 (1009 men, age 64.4 ± 11.5 years old) consecutive patients with the first AMI within 12 hours of the onset of symptoms from Baba Memorial Hospital, Tane General Hospital, Ikuwakai Memorial Hospital, Osaka City University, Osaka and Tsukazaki Memorial Hospital, Himeji, Hyougo, Japan. The diagnosis of AMI was determined by the presence of >30 minutes of continuous chest pain, ST-segment elevation 2.0 mm on 2 contiguous electrocardiographic leads, and more than a threefold increase in serum creatine kinase levels. The study protocol was approved by the Ethics Committee of each hospital.

We also obtained written informed consent from all the participants before performing coronary angiography.

### Analysis of clinical history and time of onset of AMI

Physical examinations were carefully conducted by our trained cardiology staff in the emergency room. In the Coronary Care Unit, the staff of each hospital carefully reviewed the patients’ detailed clinical history by interview and identified the time of onset. In this study, we defined cases when AMI was developed during bed rest or while sitting as “onset at rest.”

### Gravitation of the moon

The distance from the center of the moon to the center of the earth was calculated with the Software. Universal gravitation of the moon was derived by G*m/d^2^ (G: universal gravitation constant: 6.67259 × 10^−11^m^3^s^−2^ kg^−1^, m: mass of the moon, d: the distance between the center of the moon and the center of the earth). The relationship between m/d^2^ and the cases of AMI were determined.

### Statistical analysis

When we do Statistical analysis, qualitative data are presented as numbers. The numbers were compared using Poisson distribution. A p value < 0.05 was considered statistically significant difference in Poisson distribution.

## Results

**Figure 2.**
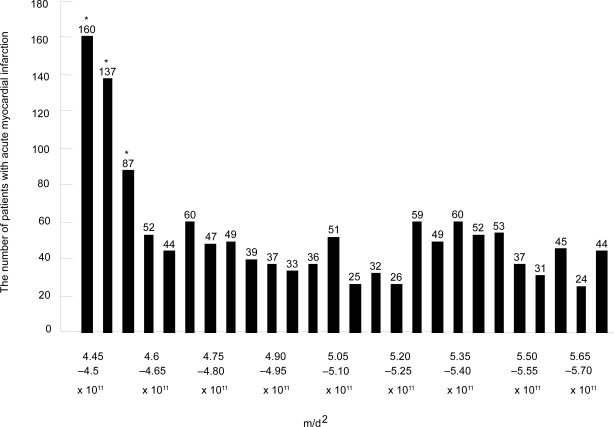
This Figure shows the relation of the gravitation of the moon and the occurrence of acute myocardial infarction. The bar graph increase by 5.0*10^9^ from the left to right. m/d^2^ was used as an index of the universal gravitation, G*M*m/d^2^. The numbers of patients with AMI are shown on the bar graph. The terms with *show the significant difference from the terms without*. G: universal gravitation constant, d: the distance from the center of the moon to the center of the earth, m:moon’s mass, M: earth’s mass.

An investigation was undertaken to determine the relation between the number of patients incurring AMI and the gravitation of the moon. Considering m/d^2^ as an index of the gravitation, an increase of AMI is found at periods of less than 4.6 × 10^11^ (Fig. 2).

## Discussion

This result suggests that the gravitation of the moon may relate to the occurrence of AMI.

Recently, the effect of the gravitation on us is getting into the news, because Lisa Randall has reported that the gravitation may have influence on our body more than we have known before. (Lisa Randall, 1999) This result may reflect her opinion.

Due to the lack of study on the relation between the motion of the moon and the occurrence of AMI, we can only speculate about the mechanism of biological clock on circadian rhythm from previous studies.

Various physiological studies on AMI have highlighted the fact that systemic physiological processes increase the intensity of the neural and hormonal system. (Middlekauff and Sontz, 1995) As some previous reports on a circadian rhythm of AMI showed (Rosing, et al. 1970; Tofler, et al. 1987), the variation of moon’s gravitation may have influence on platelet activity and fibrinolytic activity.

According to recent studies, all mammalian cells seem to possess internal biological clocks. (Dunlap, 1999) There seem to be three major components of biological clocks. These are (1) input signals, such as light in the case of the mammalian eye or hormonal factors for peripheral tissues; (2) the clock mechanism itself; and (3) the output genes. (Balsalobre, et al. 2000; Gekakis, et al. 1998) The gravitation of the moon may also regulate the cardiovascular system via internal biological clock genes as well as input signals.

Biological clock oscillation is a basic quality of human physiology and may determine the feature of cardiovascular risk.

In conclusion, because there is significant biological clock oscillation in cardiovascular events, these characteristics and the motion of the moon should be taken into account when making decisions about the treatment and prevention of ischemic heart disease. The precise mechanism of the relationship between the gravitation of the moon and cardiovascular system is not yet clearly defined. Further investigations are needed.
